# SARS-CoV-2 Seroprevalence in a Rural and Urban Household Cohort during First and Second Waves of Infections, South Africa, July 2020–March 2021

**DOI:** 10.3201/eid2712.211465

**Published:** 2021-12

**Authors:** Jackie Kleynhans, Stefano Tempia, Nicole Wolter, Anne von Gottberg, Jinal N. Bhiman, Amelia Buys, Jocelyn Moyes, Meredith L. McMorrow, Kathleen Kahn, F. Xavier Gómez-Olivé, Stephen Tollman, Neil A. Martinson, Floidy Wafawanaka, Limakatso Lebina, Jacques du Toit, Waasila Jassat, Mzimasi Neti, Marieke Brauer, Cheryl Cohen, for the PHIRST-C Group

**Affiliations:** National Institute for Communicable Diseases of the National Health Laboratory Service, Johannesburg, South Africa (J. Kleynhans, S. Tempia, N. Wolter, A. von Gottberg, J.N. Bhiman, A. Buys, J. Moyes, W. Jassat, M. Neti, C. Cohen);; University of the Witwatersrand, Johannesburg (J. Kleynhans, S. Tempia, N. Wolter, A. von Gottberg, J.N. Bhiman, J. Moyes, C. Cohen);; Centers for Disease Control and Prevention, Atlanta, Georgia, USA (S. Tempia, M.L. McMorrow);; Influenza Program, Centers for Disease Control and Prevention, Pretoria, South Africa (M.L. McMorrow);; MRC/Wits Rural Public Health and Health Transitions Research Unit (Agincourt), University of the Witwatersrand, Johannesburg (K. Kahn, F.X. Gómez-Olivé, S. Tollman, F. Wafawanaka, J. du Toit);; Perinatal HIV Research Unit, University of the Witwatersrand, Johannesburg (N.A. Martinson, L. Lebina);; Johns Hopkins University Center for TB Research, Baltimore, Maryland, USA (N.A. Martinson);; Ampath Pathology, Pretoria (M. Brauer)

**Keywords:** COVID-19, coronavirus disease, SARS-CoV-2, severe acute respiratory syndrome coronavirus 2, coronaviruses, viruses, respiratory infections, South Africa, seroprevalence, infection–case ratio, infection–hospitalization ratio, infection–fatality ratio, zoonoses

## Abstract

Severe acute respiratory syndrome coronavirus 2 (SARS-CoV-2) infections may be underestimated because of limited access to testing. We measured SARS-CoV-2 seroprevalence in South Africa every 2 months during July 2020–March 2021 in randomly selected household cohorts in 2 communities. We compared seroprevalence to reported laboratory-confirmed infections, hospitalizations, and deaths to calculate infection–case, infection–hospitalization, and infection–fatality ratios in 2 waves of infection. Post–second wave seroprevalence ranged from 18% in the rural community children <5 years of age, to 59% in urban community adults 35–59 years of age. The second wave saw a shift in age distribution of case-patients in the urban community (from persons 35–59 years of age to persons at the extremes of age), higher attack rates in the rural community, and a higher infection–fatality ratio in the urban community. Approximately 95% of SARS-CoV-2 infections were not reported to national surveillance.

The first laboratory-confirmed case of coronavirus disease (COVID-19) in South Africa was reported on March 5, 2020, and the country has since experienced 2 waves of COVID-19, the first peaking in July 2020 and the second in January 2021 (*1*). Across Africa, the second wave was more severe than the first (*2*), and specifically in South Africa, higher weekly incidence, hospitalizations, and deaths were reported for the second wave, compared with the first (*3*–*5*). The second wave in South Africa was coupled with the emergence of a new variant of severe acute respiratory syndrome coronavirus 2 (SARS-CoV-2), B.1.351, also known as 501Y.V2 or Beta (*6*).

South Africa reported >1.6 million laboratory-confirmed cases by mid-May 2021 (*3*), but many cases go undiagnosed because of mild or absent symptoms or the lack of (or reluctance to access) care or testing. Data on the proportion of persons with serologic evidence of prior SARS-CoV-2 infection are critical to assess infection rates, calculate infection–hospitalization ratios (IHRs) and infection–fatality ratios (IFRs), compare infection prevalence between waves of infection and to guide public health responses (*7*). SARS-CoV-2 seroprevalence is higher in close contacts of case-patients and at-risk healthcare workers and lower in persons <20 years of age or >65 years of age, with no differences based on sex (*8*). Whether HIV infection increases the risk for SARS-CoV-2 infection is still unclear, and results from studies thus far have varied (*9*,*10*).

We describe the seroprevalence of SARS-CoV-2 in 2 household cohorts in a rural and an urban community at 5 timepoints from July 2020 to March 2021, during 2 epidemic waves. We compare disease prevalence between the first and second wave by comparing the seroprevalence by wave to reported laboratory-confirmed infections, hospitalizations, and deaths within the respective districts.

## Methods

### Study Population

We conducted a prospective study on a randomly selected household cohort in a rural community (Agincourt, Ehlanzeni District, Mpumalanga Province) and an urban community (Jouberton, Dr. Kenneth Kaunda District, North West Province) as part of the Prospective Household Study of SARS-CoV-2, Influenza, and Respiratory Syncytial Virus Community Burden, Transmission Dynamics, and Viral Interaction (PHIRST-C) study in South Africa. Methods for the cohort study are detailed in the Appendix). Recruitment to this study began in July 2020, and follow-up will continue through August 2021. Households that previously participated in the PHIRST study during 2016–2018 (*11*,*12*) and additional randomly selected households were eligible. Households with >3 household members of any age were enrolled if >80% of members consented.

The study was approved by the University of the Witwatersrand Human Research Ethics Committee (reference no. 150808). The US Centers for Disease Control and Prevention relied on local clearance (Institutional Review Board approval no. 6840).

### Seroprevalence

We collected baseline data and blood (blood draw [BD] 1) at enrollment (July 20–September 17, 2020) and every 2 months thereafter: BD2, September 21–October 10; BD3, November 23–December 12, 2020; BD4, January 25–February 20, 2021; and BD5, March 22–April 11, 2021). We confirmed HIV status from medical records (if a person was HIV-infected) and by using a rapid test for participants with unknown or self-reported negative status. We determined previous SARS-CoV-2 infection by using the Roche Elecsys anti-SARS-CoV-2 assay (Roche Diagnostics, https://www.roche.ch/en/standorte/rotkreuz.htm) to detect antibodies against the SARS-CoV-2 nucleocapsid protein. We performed the assay on the Cobas e601 instrument (Roche Diagnostics), and we considered a cutoff index (COI) >1.0 as an indication of prior infection (i.e., seropositivity). We performed data analysis in Stata 14 (StataCorp, https://www.stata.com) (*13*). We adjusted seroprevalence estimates for sensitivity and specificity, as previously described (*14*), on the basis of the manufacturers’ reported 99.5% sensitivity and 99.8% specificity (*15*). We obtained seroprevalence 95% credible intervals (CrIs) by using Bayesian inference with 10,000 posterior draws (*14*). We used Pearson’s χ^2^ test to assess the statistical significance of differences in SARS-CoV-2 seropositivity between the 2 communities and across BDs, waves of infection, and HIV status.

### Calculation of Infection–Case Ratio, Infection–Hospitalization Ratio, and Infection–Fatality Ratio by Wave of Infection

**Figure 1:**
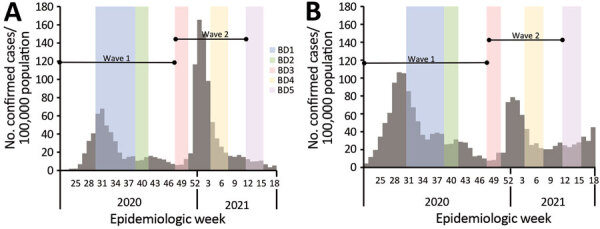
Timing of blood collection and weekly incidence of severe acute respiratory syndrome coronavirus 2 infection in the rural community district (A) and the urban community district (B), South Africa, March 2020–March 2021. BD, blood draw.

To assess the prevalence of SARS-CoV-2 and compare the severity of illness between the 2 waves, we performed an ecologic study comparing estimated number of infections on the basis of seroprevalence in our cohort study to reported number of cases, hospitalizations, and in-hospital and excess deaths in the same district for each wave. We calculated the age- and sex-adjusted total number of infections, laboratory-confirmed cases, hospitalizations, deaths, infection–case ratio (ICR) (i.e., number of infections compared with laboratory-confirmed cases), IHR, and in-hospital and excess deaths IFR (Appendix) for the first (March 1–November 21, 2020) and second (November 22, 2020–March 27, 2021) wave of infection (Figure 1). 

### Comparison of Cases between First and Second Wave of Infection

We compared characteristics of participants who showed seroconversion during the first and second wave of infections by using unconditional logistic regression. We compared participants who showed seroconversion in wave 1 (BD3) with those who showed seroconversion in wave 2 (BD5, excluding BD3 seroconversions). For this analysis, we only included participants with a BD3 and BD5 paired serum sample. For the multivariable model, we assessed all variables that were significant at p<0.2 on univariate analysis and dropped nonsignificant factors (p>0.05) with manual backward elimination. We also compared the site, age, sex, and HIV status of persons with a BD 3+5 pair with those without a BD 3+5 pair by using logistic regression.

### Persistence of SARS-CoV-2 Antibodies

For participants with 5 serum samples collected and who showed seroconversion during BD2 to BD5, we plotted COI values with the BD at which seroconversion took place as point 0. For participants who were seropositive at baseline, we plotted COI results from each BD. We calculated mean COI and the exact 95% CI at each point by using the Clopper–Pearson method. We assessed percentage of participants with COI >1 at each subsequent BD as number of participants with COI >1 divided by total number of participants who showed seroconversion during BD2 to BD5 with a serum sample at the timepoint.

## Results

### Study Population

In the rural community, we approached 185 households, 118 (64%) were enrolled, and 641/692 (92%) of household members consented, agreed to participate, or both. In the urban community, 352 households were approached, 114 (32%) enrolled, and 570/607 (93%) of household members consented, agreed to participate, or both. In both communities, the percentage of children, women or girls, and unemployed persons included in the cohort were higher than in district census data (Appendix). Median age was 13 (interquartile range 7–29) and 21 (interquartile range 10–43) years, and HIV prevalence was 14% (95% CI 11%–17%) in the rural community and 18% (95% CI 14%–21%) in the urban community.

### Seroprevalence

**Figure 2:**
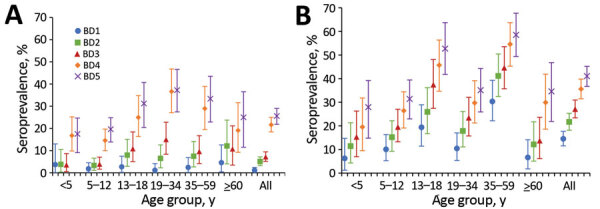
Seroprevalence of severe acute respiratory syndrome coronavirus 2 at each blood collection, by age group, in a rural community (A) and an urban community (B), South Africa, March 2020–March 2021.

Most (83% [n = 553]) participants who lived in the rural community and most (83% [n = 499]) who lived in the urban community had both BD3 and BD5 blood collected (Appendix). Seroprevalence, adjusted for assay sensitivity and specificity, in the rural community was lower at BD1 than in the urban community (1% [95% CrI 0%–2%] vs. 15% [95% CrI 12%–18%]; p<0.001), increasing after the first wave of infections (at BD3) to 7% (95% CrI 5%–9%) in the rural community and 27% (95% CrI 23%–31%) in the urban community (p<0.001) (Figure 2; Appendix). After the second wave (BD5), seroprevalence increased to 26% (95% CrI 22%–29%; p<0.001) in the rural community and to 41% (95% CrI 37%–45%; p<0.001) in the urban community (Appendix).

At BD5, seroprevalence was highest in the 19–34 years age group (37% [95% CrI 28%–47%]) in the rural community and the 35–59 years age group (59% [95% CrI 49%–68%]) in the urban community (Appendix). The seroprevalence was lowest in children <5 years of age, 18% (95% CrI 10%–26%) in the rural community and 28% (95% CrI 17%–41%) in the urban community.

At BD5, SARS-CoV-2 seroprevalence was similar between HIV-infected and HIV-uninfected participants (Appendix). Persons who were HIV-positive were not more likely to be seropositive (adjusted odds ratio 1.0 [95% CI 0.7–1.5]).

### Infection–Case Ratio, Infection–Hospitalization Ratio, and Infection–Fatality Ratio by District and Wave of Infection

**Figure 3:**
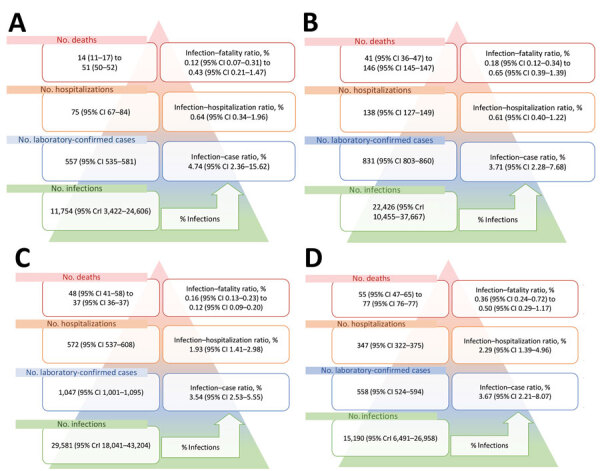
Age- and sex-standardized number of severe acute respiratory syndrome coronavirus 2 infections, laboratory-confirmed diagnoses, hospitalizations, and deaths per 100,000 population in a rural community during infection wave 1 (A) and wave 2 (B) and an urban community during infection wave 1 (C) and wave 2 (D), South Africa, March 2020–March 2021. CrI, credible interval.

**Figure 4:**
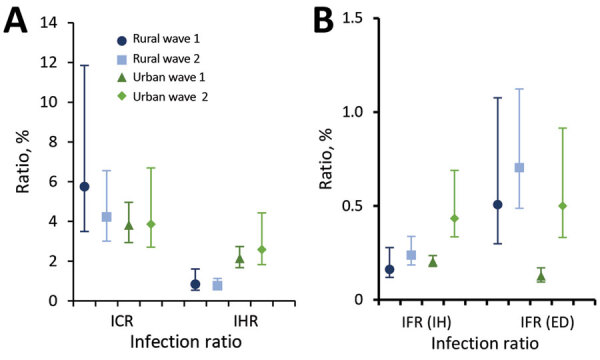
Severe acute respiratory syndrome coronavirus 2 infection–case and infection–hospitalization ratios (A) and in-hospital and excess deaths infection-fatality ratios (B) in a rural and urban community during the first and second wave of infections, South Africa, March 2020–March 2021. Vertical lines represent 95% CIs. Wave 1: March 1–November 21, 2020. Wave 2: November 2020 22–March 27, 2021. ED, excess deaths; ICR, infection–case ratio; IFR, infection–fatality ratio; IH, in-hospital; IHR, infection–hospitalization ratio.

During the first wave of infections (BD3) the age- and sex-adjusted seroprevalence at the rural site was 11.75% (95% CrI 3.42%–24.60%), resulting in an ICR of only 4.74% (95% CI 2.36%–15.62%). We observed a 0.64% (95% CI 0.34%–1.96%) IHR and an in-hospital IFR of 0.12% (95% CI 0.07%–0.31%) and an excess deaths IFR of 0.43% (95% CI 0.21%–1.47%) (Figure 3, 4).

The seroprevalence in the rural community was 22.43% (95% CrI 10.46%–37.67%) for the second wave. The ICR was 3.71% (95% CI 2.28%–7.68%), IHR was 0.61% (95% CI 0.40%–1.22%), in-hospital IFR was 0.18% (95% CI 0.12%–0.34%), and excess deaths IFR was 0.65% (95% CI 0.39%–1.39%) (Figure 3, 4).

In the urban community, the seroprevalence at BD3 was 29.58% (95% CrI 18.04%–43.20%). We found a 3.54% (95% CI 2.53%–5.55%) ICR and 1.93% (95% CI 1.41%–2.98%) IHR. The in-hospital IFR was 0.16% (95% CI 0.13%–0.23%) and excess deaths IFR was 0.12% (95% CI 0.09%–0.20%) (Figure 3, 4). During the second wave, the seroprevalence in the urban community was 15.19% (95% CrI 6.49%–26.96%), resulting in an ICR estimate of 3.67% (95% CI 2.21%–8.07%), an IHR of 2.29% (95% CI 1.39%–4.96%), an in-hospital IFR of 0.36% (95% CI 0.24%–0.72%), and an excess deaths IHR of 0.50% (95% CI 0.29%–1.17%) (Figure 3, 4). These estimates standardized to World Health Organization world population estimates are shown in Appendix Figure 2.

### Comparison of Case-Patients between First and Second Wave of Infection

Compared with the urban community, persons in the rural community who showed seroconversion were 4.7 (95% CI 2.9–7.6) times more likely to show seroconversion during the second wave. Compared with persons 35–59 years of age, persons 5–12 years of age were 2.1 (95% CI 1.1–4.2) times more likely to show seroconversion in the second wave and persons >60 years of age were 2.8 (95% CI 1.1–7.0) times more likely to show seroconversion in the second wave (Table). When we stratified the analysis by site, this association was only detected in the urban community (Appendix). Persons who did not have a BD 3+5 pair were more likely to be <5 or 19–34 years of age (Appendix).

**Table:** Comparison of participants with detectable SARS-CoV-2 antibodies after the first wave (blood draw 3) and second wave (blood draw 5), South Africa, July 2020–April 2021*

Characteristic	Infected in wave 1, no. (%)	Infected in wave 2, no. (%)	Univariate OR (95% CI)	Multivariable aOR (95% CI)
Site				
Rural	40/140 (29)	100/140 (71)	**4.9 (3.1–7.8)**	**4.7 (2.9–7.6)**
Urban	139/210 (66)	71/210 (34)	Referent	Referent
Sex				
M	65/123 (53)	58/123 (47)	Referent	
F	114/227 (50)	113/227 (50)	1.1 (0.7–1.7)	
Age group, y				
<5	9/25 (36)	16/25 (64)	**3.2 (1.3–8.0)**	2.7 (1.0–7.2)
5–12	30/74 (41)	44/74 (59)	**2.6 (1.4–4.9)**	**2.1 (1.1–4.2)**
13–18	36/64 (56)	28/64 (44)	1.4 (0.7–2.7)	1.3 (0.6–2.6)
19–34	34/67 (51)	33/67 (49)	1.7 (0.9–3.3)	1.3 (0.7–2.6)
35–59	59/92 (64)	33/92 (36)	Referent	Referent
>60	11/28 (39)	17/28 (61)	**2.8 (1.2–6.6)**	**2.8 (1.1–7.0)**
HIV status				
Negative	139/271 (51)	132/271 (49)	1.0 (0.6–1.7)	
Positive	35/68 (51)	33/68 (49)	Referent	
CD4 count, cells/μL				
>200	28/54 (52)	26/54 (48)	1.9 (0.2–21.7)	
<200	2/3 (67)	1/3 (33)	Referent	
Viral load, copies/mL				
<1,000	28/51 (55)	23/51 (45)	Referent	
>1,000	2/7 (29)	5/7 (71)	3.0 (0.5–17.2)	
Other underlying illness‡				
No	161/316 (51)	155/316 (49)	1.1 (0.5–2.2)	
Yes	18/34 (53)	16/34 (47)	Referent	
Body mass index category				
Underweight	10/22 (45)	12/22 (55)	1.5 (0.6–3.9)	
Normal weight	65/141 (46)	76/141 (54)	1.5 (0.9–2.5)	
Overweight	46/84 (55)	38/84 (45)	1.1 (0.6–1.9)	
Obese	58/103 (56)	45/103 (44)	Referent	
Currently smoking‡				
No	109/190 (57)	81/190 (43)	Referent	
Yes	20/36 (56)	16/36 (44)	1.1 (0.5–2.2)	
Alcohol use‡				
No	88/167 (53)	79/167 (47)	2.0 (1.1–3.8)	
Yes	41/59 (69)	18/59 (31)	Referent	
Employment status§				
Unemployed	70/128 (55)	58/128 (45)	1.9 (0.5–6.4)	
Student	9/13 (69)	4/13 (31)	Referent	
Employed	25/41 (61)	16/41 (39)	1.4 (0.4–5.5)	

*Includes all participants with blood draw 3 and 5 serum pairs and who showed seroconversion at either draw. Bold indicates a statistically significant difference. aOR, adjusted odds ratio; OR, odds ratio; SARS-CoV-2, severe acute respiratory syndrome coronavirus 2. †Self-reported history of asthma, lung disease, heart disease, stroke, spinal cord injury, epilepsy, organ transplant, immunosuppressive therapy, organ transplantation, cancer, liver disease, renal disease, or diabetes. ‡Among persons >15 years of age. §Among persons >18 years of age.

### Persistence of SARS-CoV-2 Antibodies

**Figure 5:**
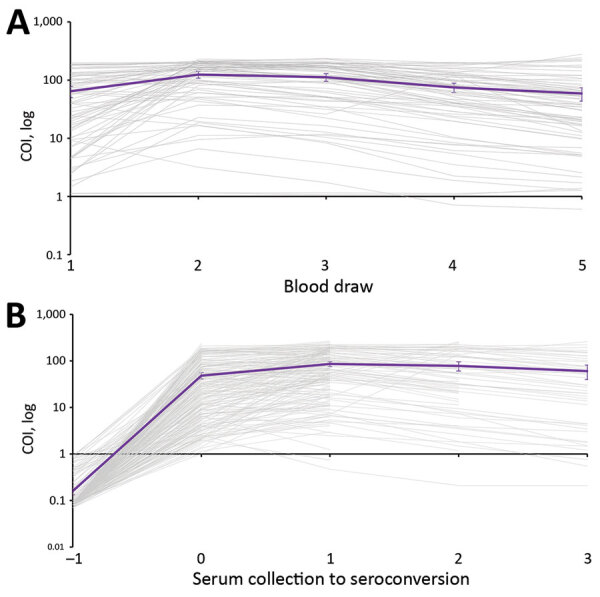
Cutoff index (COI) on Roche Elecsys (Roche Diagnostics, https://www.roche.ch/en/standorte/rotkreuz.htm) anti–severe acute respiratory syndrome coronavirus 2 assay for persons with blood draws 1–5 samples who were seropositive at baseline (A) or showed seroconversion during blood draws 2–5, South Africa, July 2020–April 2021. Purple line indicates mean COI with 95% CIs. COI values in panel B are aligned to first draw before seroconversion, COI, cutoff index.

Of the 72 participants who were seropositive at BD1 and with BDs 1–5 samples collected, 99% (71/72) still had a COI >1 by BD5. The mean COI at baseline for seropositive participants was 64, which increased to 125 at BD2 and dropped to 59 at BD5 (Figure 5, panel A). The participant who no longer had detectable SARS-CoV-2 antibodies at BD5 had a starting COI of 9. Of the 210 participants with BD 1–5 samples, 99% (167/169), 99% (70/71%), and 93% (41/44) still had a COI >1 in the first, second, and third BD after initial seroconversion, respectively (Figure 5, panel B). The participants who seroreverted had starting COIs ranging from 2 to 6, and none showed seroconversion again after reversion during the study period. The mean COI at the point of seroconversion was 48, which increased to 86 at the first BD after seroconversion and reduced to 61 at the third BD after seroconversion.

## Discussion

We assessed SARS-CoV-2 seroprevalence in 1,211 persons living in 2 diverse communities in South Africa and show that laboratory-confirmed cases reported from study districts greatly underestimate the actual prevalence of SARS-CoV-2 infections. At baseline, seroprevalence was 1% and 15%, increasing to 7% and 27%, respectively, after the first wave, by March 2021. After the second epidemic wave, seroprevalence was 26% in the rural community and 41% in the urban community. The highest seroprevalence was 59% in adults 35–59 years of age in the urban community, and the lowest was 18% in rural community children <5 years of age. During the second wave, compared with the first wave, the rural site was more affected, and infections in the second wave more likely affected children 5–12 and adults >60 years of age in the urban community. In the urban community, IFR was higher in the second wave (0.36%–0.50%) compared with the first (0.12%–0.16%), and numbers of infections were lower, suggesting possible increased severity associated with the emergence of novel variant B.1.351. Most persons who showed seroconversion maintained detectable SARS-CoV-2 antibodies in subsequent serum samples.

Low seropositivity was observed at the rural site at baseline, and seroprevalence remained low after the first wave of infections, reaching 7%, which was considerably lower than the 27% at the urban site at the same time. This observation could be related to the relatively isolated location and lower population density in the rural community compared with more densely populated urban community. Seroprevalence in the rural site increased to 26% after the second wave of infections within the district. This increase could have been attributable to possible increased transmissibility of the B.1.135 lineage that was circulating in the second wave (*16*), as well as additional transmission networks in the community during the December holiday period, when largescale urban-to-rural migration takes place as persons return home for year-end holidays. The urban site had fewer seroconversions in the second wave compared with the first, which may be attributable to existing immunity among persons in the community after the first wave. As seen in previous studies (*8*), adults had the highest seroprevalence levels, although a relatively high seroprevalence of 18% and 28% persisted in children <5 years of age at the rural and urban community, respectively.

When comparing the characteristics of persons infected in the second wave to those infected in the first, persons infected in the second wave were more likely to be from the rural site and to be <13 or >60 years of age, compared with persons 35–59 years of age. The shift in age groups affected was only detected in the urban community, possibly because of the large number of adults infected during wave 1, whereas the number of infections in wave 1 in the rural community was lower.

A study conducted among blood donors in South Africa during the second wave found a seroprevalence of 32%–63% in 5 provinces of South Africa that have both rural and urban communities (W. Sykes et al., unpub. data, https://doi.org/10.21203/rs.3.rs-233375/v1). In our study, we observed a seroprevalence in adults ranging from 25% to 37% in rural households, and from 35% to 59% in urban households, suggesting that seroprevalence is heterogeneous between communities. In Kenya, the seroprevalence in blood donors during the country’s first wave of infections was 4% and was also higher in urban communities (*17*). In a population-level household serosurvey conducted in Zambia during their first wave of infections, 11% of persons had evidence of SARS-CoV-2 infection (*18*).

Based on our estimates, only 4%–6% of cases were laboratory-confirmed, suggesting that substantially higher prevalence of infection was ascertained through serologic testing and that the differences may have been greater in the urban community than the rural community; however, more extensive studies are needed to assess whether this observation is consistent in other areas. Compared with the urban community, the rural community had less than half the rate of hospitalization (0.6% vs. 2.0%). These observations may be attributable to differences in referral and testing policies, health-seeking behavior, and access to care, as well as differences in circulating lineages within these districts.

A study comparing the severity of the first and second waves of infections in South Africa in hospitalized patients found a higher mortality rate in the second wave, compared with the first (*19*). At the urban site, the IFR was higher in the second wave (0.36%–0.50%) compared with the first (0.12%–0.16%), although no differences were observed in IHR between the 2 waves. The lower overall number of infections in the second wave in this site means that our finding of increased mortality is unlikely to be related to pressure on health services. The increased severity of the second wave may be related to increased severity of the B.1.135 variant, but further studies are needed to confirm this relation. The excess death IFR during the first wave in the urban site was smaller than the in-hospital IFR. This difference may be attributable to uncertainty on the process for excess death estimation, or that the 85% contribution of COVID-19 to excess deaths was an underestimation within the province. However, the in-hospital IFR followed the same trend of increase between wave 1 and 2 (0.16% to 0.36%). Although no significant increase was observed between the IFR in the rural community between wave 1 and 2, the excess (maximum) IFR estimate (0.43%) was already high in wave 1, similar to the IFR for wave 2 in the urban community (0.50%). Considering the lower IHR in the rural community for both waves compared with the urban community, this observation may point toward lack of access to care or delays in seeking care. In-hospital SARS-CoV-2 mortality rates have previously also been shown to be higher in Mpumalanga Province where the rural community is located (*19*).

Our first wave in-hospital IFR estimates (0.12% rural, 0.16% urban) were similar to the age-adjusted 0.15% reported from India for the first wave of laboratory-confirmed deaths from SARS-CoV-2 infection (*20*). In addition, our first wave excess death IFR was higher in the rural (0.431%) and lower in the urban (0.12%) community compared with the age-adjusted 0.28% IFR excess deaths reported from Brazil during their first wave of infections (*21*).

Although previous studies have shown that antibodies against the SARS-CoV-2 nucleocapsid wanes more quickly than those against the spike protein (*22*,*23*), 93% of persons who showed seroconversion at BD2 still had detectable nucleocapsid antibodies 6 months later. Direct antigen-sandwich format assays, such as the Roche anti-N assay used in our study, have been found to reliably detect antibodies in longitudinal samples (*24*). Of the few that seroreverted in this timeframe, the starting COI was low.

We did not observe a difference in SARS-CoV-2 seroprevalence in HIV-infected and HIV-uninfected persons at either site. Although our sample size was too small to detect small differences in a stratified analysis, we also did not observe a signal when using logistic regression. Because HIV causes immune suppression, a concern exists that HIV-infected persons may be more susceptible to SARS-CoV-2 infection (*9*,*10*). Although HIV infection may not increase susceptibility to infection, it has been demonstrated to be a risk factor for having onset of severe COVID-19 and death after infection (*9*,*25*).

Our study is limited by a small sample size, reducing the power for accurate seroprevalence estimates in small age strata, and inclusion of only 2 geographic sites, and therefore may not be representative of other districts and provinces in South Africa. Because we used seroprevalence to estimate infections by wave, we could have missed reinfections in the second wave. Based on data from the same cohort, these reinfections occurred in only a small portion (3%) of the cohort (C. Cohen et al., unpub. data, https://doi.org/10.1101/2021.07.20.21260855) and would have had a negligible influence on the infection ratios. ICR, IHR, and IFR formed part of an ecologic analysis, which is inherently prone to biases. Excess deaths in the first wave may be underestimated because the reporting period only started in June. Transmission dynamics within our cohort may not be similar to the transmission dynamics within the district. Seventeen percent of persons did not have a BD 3+5 pair, and bias could have been introduced if the seroprevalence were different for those without a BD 3+5 blood pair. Ongoing follow-up of this cohort will track future infections and monitor antibody waning, and compare these data to laboratory-confirmed infections and symptoms from twice-weekly follow-up. 

A strength of our study is the collection of samples from prospectively followed-up persons from randomly selected households within the study communities and inclusion of persons of all ages. As a longitudinal study, our study provides the advantage of serial comparisons of antibody responses in relation to reported laboratory-confirmed SARS-CoV-2 infections within the community through 2 successive SARS-CoV-2 waves.

We estimate that ≈95% of SARS-CoV-2 infections in these 2 communities were not laboratory-confirmed and reported to the national surveillance system, which has major implications for contact tracing and isolation and other measures to contain infection. We observed heterogeneity between seroprevalence estimates based on pandemic wave, community, and age group, indicating the need for ongoing studies that include diverse settings.

Additional Members of the PHIRST-C Group who Contributed to this Manuscript Kgaugelo Patricia Kgasago, Linda de Gouveia, Maimuna Carrim, Mignon du Plessis, Retshidisitswe Kotane, and Tumelo Moloantoa.

Appendix Additional information about longitudinal SARS-CoV-2 seroprevalence in a rural and urban community household cohort during first and second waves of infections, South Africa, July 2020–March 2021.
