# Supplementary material for: SARS-CoV-2 Seroprevalence in a Rural and Urban Household Cohort during First and Second Waves of Infections, South Africa, July 2020–March 2021
Source: Emerg Infect Dis. 2021 Dec;27(12):3020–9. doi: 10.3201/eid2712.211465 (PMC8632160; doi:10.3201/eid2712.211465)
Supplement: Appendix — Additional information about longitudinal SARS-CoV-2 seroprevalence in a rural and urban community household cohort during first and second waves of infections, South Africa, July 2020–March 2021. [file 21-1465-Techapp-s1.pdf]

Article DOI: https://doi.org/10.3201/eid2712.211465

SARS-CoV-2 Seroprevalence in a Rural and Urban Household Cohort during First and Second Waves of Infections, South Africa, July 2020–March 2021

Appendix

Supplementary Methods

Study Population

PHIRST-C (A Prospective Household study of SARS-CoV-2, Influenza, and Respiratory Syncytial virus community burden, Transmission dynamics and viral interaction in South Africa) is a continuation from PHIRST (A Prospective Household observational cohort study of Influenza, Respiratory Syncytial virus and other respiratory pathogens community burden and Transmission dynamics in South Africa) (*1*). PHIRST-C is conducted in two communities with established influenza-like illness and pneumonia surveillance sites (Appendix Figure 1).

South Africa consists of nine provinces, which are divided into 52 districts, which form the second level of administration. Districts are further divided into municipalities. The rural site is located in the Bushbuckridge Municipality, Ehlanzeni District, Mpumalanga Province and forms part of a health and sociodemographic surveillance site (HDSS) at the Medical Research Council (MRC)/University of Witwatersrand Rural Public Health and Health Transitions Research Unit, Agincourt. During PHIRST, two of the 29 villages within the HDSS were selected according to convenience (proximity and burden of other studies within the site) each year between 2016–2018. A random selection of ≈50 households with a household size of 3 or more were approached for enrolment. The urban site is located in the Jouberton Township, Matlosana Municipality, Dr Kenneth Kaunda District, North West Province. A list of 450 global positioning system (GPS) coordinates were generated using Google Earth. Study staff approached the nearest house within 30 m of the coordinate point for enrolment. Approximately 50 households were enrolled each year during 2016–2018. At both sites, households were eligible if they consisted of 3 or more household members

(sharing at least four meals a week). All household members were approached for inclusion in the study, and households were eligible for inclusion if >80% of members consented to be enrolled.

For PHIRST-C, all households who participated in PHIRST from the urban site, and households from the 2017 and 2018 cohort in the rural site were approached for enrolment. To supplement the sample size, additional households at each site were approached using the same methods as for PHIRST. Villages in the rural site were restricted to those four used in 2017 and 2018 of PHIRST. Informed consent was obtained from participating adults or a parent/guardian for children <18 years of age. In addition to parent/guardian consent, assent was also obtained from children aged 7–17 years. When performing rapid HIV tests, both pre- and post-test counselling was provided, including referral to care for those who tested positive.

The Bushbuckridge Municipality within the Ehlanzeni District is considered as predominantly rural, with main industries being agriculture and tourism (*2*). The Ehlanzeni District has a population of 1,828,738, with sixty percent of the district's population being >18 years of age (Appendix Table 1). The City of Matlosana Municipality is considered to be 88.2% urban, and is located in the Dr Kenneth Kaunda District, which has a population of 797,715 (*3*). Sixty-five percent of the district population is aged >18 years (Appendix Table 1). At both sites, the percentage of children, females, and unemployed individuals were higher in the cohort than in the district (Appendix Table 1).

Calculation of Infection-Case Ratio (ICR), Infection-Hospitalization Ratio (IHR), and Infection-Fatality Ratio (IFR) by Wave of Infection

We calculated the age-adjusted total number of infections, laboratory-confirmed cases, hospitalizations, deaths, ICR (number of infections compared to laboratory-confirmed cases), IHR and in-hospital and excess death IFR for each wave of infection as described in below equations. We defined the first wave as March 1, 2020 (week 11) to November 21, 2020 (week 47), coinciding with the first case of SARS-CoV-2 reported in South Africa, and ending the week before BD3 started. We defined the second wave as November 22, 2020 (week 48) to March 27, 2021 (week 11), starting directly after the defined wave 1 period, and ending the week before BD5 started. Due to differences between the sex ratios of our cohort and the district population, the infection estimates were also adjusted for sex. Data sources used in these calculations are described in the next section. Age standardized estimates for the selected endpoints for each wave were obtained as follows:

$$Infections = \frac{\sum_i (s_i \times SAp_i)}{\sum_i (SAp_i)} \times 100{,}000$$

Where s_i is the seroprevalence in the cohort in the respective community and wave for age and sex group *i* and $p_{i(SA)}$ is the South African population for age and sex group *i*. Calculated for wave 1 as seroprevalence at blood draw 3, and for wave 2 as seroprevalence at blood draw 5, excluding those who seroconverted at blood draw 3. Estimates only included participants with a blood draw 3 and 5 pair, and adjusted for sensitivity and specificity of test (*4*).

$$Laboratory - confirmed\ cases = \frac{\sum_i \left((c_i \div p_{i(d)}) \times p_{i(SA)} \right)}{\sum_i \left(p_{i(SA)} \right)} \times 100{,}000$$

Where c_i is the number of laboratory-confirmed cases (RT-PCR and antigen-based tests) from the respective district reported to the NMCSS (wave 1: 3 March – 21 November 2020, wave 2: 22 November 2020 – 27 March 2021) in age group *i*, $p_{i(d)}$ is the district population for age group *i* and $p_{i(SA)}$ is the South African population for age group *i*.

$$Hospitalisations = \frac{\sum_i \left((h_i \div p_{i(d)}) \times p_{i(SA)} \right)}{\sum_i \left(p_{i(SA)} \right)} \times 100{,}000$$

Where h_i is the number of hospitalizations from the respective district reported to COVID-19 Sentinel Hospital Surveillance (DATCOV, wave 1: 5 March – 21 November 2020, wave 2: 22 November 2020 – 27 March 2021) (*5*) in age group *i*, $p_{i(d)}$ is the district population for age group *i* and $p_{i(SA)}$ is the South African population for age group *i*.

$$In - hospital\ deaths = \frac{\sum_i \left((d_i \div p_{i(d)}) \times p_{i(SA)} \right)}{\sum_i \left(p_{i(SA)} \right)} \times 100{,}000$$

Where d_i is the number of in-hospital deaths from the respective districts reported to DATCOV (wave 1: 5 March–21 November 2020, wave 2: 22 November 2020–27 March 2021) (*5*) in age group *i*, $p_{i(d)}$ is the district population for age group *i* and $p_{i(SA)}$ is the South African population for age group *i*.

$$Excess\ deaths = \frac{(ExD\ x\ 0.85)\ \times\ p_{(SA)}}{p_{(SA)}} \times 100{,}000$$

Where *ExD* is the rate of provincial excess deaths adjusted to the South African population reported by South African Medical Research Council (rural wave 1: 28 June–21 November 2020, urban wave 1: 21 June–21 November 2020, both communities wave 2: 22

November 2021–26 March 2021) (*6*), and $p_{(SA)}$ is the total South African population. According to estimates only 85% of excess deaths are attributable to COVID-19 (*6*).

For infections, hospitalizations, in-hospital (minimum) and excess (maximum) deaths, 95% CIs were calculated using the Clopper-Pearson method with a Poisson distribution.

$$Infection - case\ ratio\ (ICR) = \frac{Laboratory - confirmed\ cases}{Infections}\ x\ 100$$

$$Infection - hospitalization\ ratio\ (IHR) = \frac{Hospitalisations}{Infections}\ x\ 100$$

$$Infection - fatality\ ratio\ (IFR) = \frac{Deaths}{Infections}\ x\ 100$$

Confidence intervals for infection ratios were calculated as ratios from the 95% confidence intervals of infection, hospitalization and death rates. For international comparisons, we repeated the calculations to standardize to the WHO standard world population (*7*), using Sprague multipliers (*8*) to expand age groups to 1-year bands, and aggregate to the age groups used in this study.

Data Sources

Population Denominators (*9*)

Population numbers for each district, by age, were obtained from the StatsSA 2020 mid-year population estimates for 2020.

Notifiable Medical Conditions Surveillance System (NMCSS) (*10*)

Reverse transcription PCR (RT-PCR) testing in South Africa to detect SARS-CoV-2 RNA started on 28 January 2020. Rapid SARS-CoV-2 antigen testing was implemented in November 2020. All laboratories in the private and public sector performing SARS-CoV-2 tests automatically feed testing data from their lab information systems to the NMCSS, from where daily and weekly reporting on national, provincial and district SARS-CoV-2 infections are performed. No serology tests are used for this reporting. Results from antigen tests may be underestimated in the NMCSS. The number of cases reported to the NMCSS in the Ehlanzeni District (rural community) and Dr Kenneth Kaunda District (urban community) was used as an estimate of reported, laboratory-confirmed SARS-CoV-2 infections. Wave 1: 3 March 1 November 2020, wave 2: 22 November 2020–27 March 2021.

COVID-19 National Hospital Surveillance (DATCOV) (*5*)

DATCOV is a national hospital surveillance system to which all hospitals where COVID-19 admissions occurred in the public and private sector in South Africa report to. The case definition includes any person admitted with a positive RT-PCR test result for SARS-CoV-2. In-hospital outcome was available for all patients. The total number of hospitalizations and in-hospital deaths in each district reported to DATCOV between 3 March –21 November 2020 (wave 1) and 22 November 2020–27 March 2021 (wave 2) was used to calculate in the IHR and minimum IFR, respectively.

South African Medical Research Council (SAMRC) Report on Weekly Deaths (*6*)

The Burden of Disease Unit at the SAMRC produces a weekly report on excess deaths in South Africa. Deaths in South Africa are registered on the Department of Home Affair's National Population Register and includes all citizens with a South African identification number. The number of excess deaths were defined as the number of all-cause deaths in that week minus the number of deaths expected for the week based on 2014–2019 trends. Excess death estimates are adjusted for incomplete reporting, and death estimates in provinces are age-standardized to the national population. Eighty-five percent of the reported excess deaths were used to calculate the maximum IHR. In both provinces, excess deaths were only available starting 21 June (urban) and 28 June 2020 (rural), and not from March as for infections and hospitalizations, therefore the period for wave 1 in the rural community was defined as 28 June –21 November 2020, and as 21 June–21 November 2020 in urban communities for wave 1. For both communities wave 2 was defined as 22 November 2021–26 March 2021. The wave-specific excess death rates were kindly provided by Professor Rob Dorrington.

Differences in SARS-CoV-2 Seroprevalence by HIV Status and Age

To assess differences in seropositivity based on HIV status, we considered seroprevalence at the fifth blood draw and compared the seroprevalence of individuals at each site and age group by HIV status using the Pearson's chi-squared test, and furthermore also compared the seroprevalence by HIV status while controlling for site, sex and age using logistic regression. We calculated power using the two-sample proportion, likelihood-ratio test for a 95% CI, to detect an odds ratio of at least 1.8, assuming a 24% SARS-CoV-2 seroprevalence and 14% HIV prevalence. In the 19–34 year old group (n = 247) we only had 33% power.

References

1. Cohen C, Kleynhans J, Moyes J, McMorrow ML, Treurnicht FK, Hellferscee O, et al.; PHIRST Group. Asymptomatic transmission and high community burden of seasonal influenza in an urban and a rural community in South Africa, 2017–18 (PHIRST): a population cohort study. Lancet Glob Health. 2021;9:e863–74. PubMed https://doi.org/10.1016/S2214-109X(21)00141-8

2. Bushbuckridge Local Municipality. Bushbuckridge Local Municipality final integrated development plan 2020/21. 2020 [cited 2021 Feb 16]. https://www.cogta.gov.za/cgta_2016/wp-content/uploads/2021/02/Bushbuckridge-Municipality.pdf

3. City of Marlosana. Matlosana spatial development framework. 2009 [cited 2021 Feb 16]. http://www.matlosana.gov.za/Documents/Policies/SPLUMA/SDF%20FRAMEWORK%202009.pdf

4. Larremore DB, Fosdick BK, Bubar KM, Zhang S, Kissler SM, Metcalf CJE, et al. Estimating SARS-CoV-2 seroprevalence and epidemiological parameters with uncertainty from serological surveys. eLife. 2021;10:e64206. PubMed https://doi.org/10.7554/eLife.64206

5. National Institute for Communicable Diseases. COVID-19 sentinel hospital surveillance update, week 18. 2021 May 14 [cited 2021 May 22]. https://www.nicd.ac.za/wp-content/uploads/2021/05/NICD-COVID-19-Weekly-Sentinel-Hospital-Surveillnace-update-Week-18-2021.pdf

6. South African Medical Research Council. Report on weekly deaths in South Africa, week 18. 2021 May 10 [cited 2021 May 22]. https://www.samrc.ac.za/reports/report-weekly-deaths-south-africa

7. Ahmad OB, Boschi-Pinto C, Lopez AD, Murray CJ, Lozano R, Inoue M. Age standardization of rates: a new WHO standard. 2001 [cited 2021 May 5]. https://www.who.int/healthinfo/paper31.pdf

8. Shryock HS, Larmon EA, Siegel JS; US Department of Commerce, Bureau of the Census. The methods and materials of demography. Washington: US Government Printing Office; 1980.

9. Statistics South Africa. Statistical release P0302: mid-year population estimates 2020. 2020 [cited 2021 May 14]. http://www.statssa.gov.za/?page_id=1854&PPN=P0302&SCH=72634

10. National Institute for Communicable Diseases. COVID-19 weekly epidemiology brief, week 19. 2021 May 22 [cited 2021 May 22]. https://www.nicd.ac.za/wp-content/uploads/2021/05/COVID-19-Weekly-Epidemiology-Brief-week-19-2021.pdf

Appendix Table 1. Comparison of characteristics between district (Ehlanzeni and Dr Kenneth Kaunda Districts) (9) and PHIRST-C cohort population, 2020, South Africa.

Characteristic	Ehlanzeni District	Rural community cohort	p-value	Dr Kenneth Kaunda Districts	Urban community cohort	p-value
Age group, y						
<5	203727/1828739 (11%)	99/668 (15%)	<0.01	75574/797716 (9%)	56/598 (9%)	0.93
5–12	319208/1828739 (17%)	210/668 (31%)	<0.01	121145/797716 (15%)	132/598 (22%)	<0.01
13–18	207344/1828739 (11%)	89/668 (13%)	0.11	82582/797716 (10%)	82/598 (14%)	0.01
19–34	491914/1828739 (27%)	108/668 (16%)	<0.01	212152/797716 (27%)	120/598 (20%)	<0.01
35–59	462982/1828739 (25%)	89/668 (13%)	<0.01	237146/797716 (30%)	122/598 (20%)	<0.01
≥60	143564/1828739 (8%)	46/668 (7%)	0.35	69117/797716 (9%)	58/598 (10%)	0.37
Female sex	961384/1828739 (53%)	427/668 (64%)	<0.01	405734/797716 (51%)	336/598 (56%)	0.01
Unemployed	187428/530865 (35%)	198/270 (73%)	<0.01	76654/257457 (30%)	202/315 (64%)	<0.01

Appendix Table 2. Number of sera collected (n) from consenting PHIRST-C participants (N) and percentage sampled during blood draws (BD) in the rural and urban community by blood draw, site, age group, March 2020 – March 2021, South Africa*

	No. (%)						
Characteristic	BD1	BD2	BD3	BD4	BD5	BD1–5	BD3,5
Rural							
Age group, y							
<5	25/102 (25)	49/102 (48)	78/102 (76)	87/102 (85)	89/102 (87)	17/102 (17)	76/102 (75)
5–12	148/215 (69)	169/215 (79)	195/215 (91)	202/215 (94)	201/215 (93)	136/215 (63)	193/215 (90)
13–18	69/94 (73)	72/94 (77)	80/94 (85)	82/94 (87)	78/94 (83)	61/94 (65)	76/94 (81)
19–34	87/117 (74)	88/117 (75)	91/117 (78)	91/117 (78)	95/117 (81)	62/117 (53)	89/117 (76)
35–59	76/91 (84)	77/91 (85)	80/91 (88)	81/91 (89)	82/91 (90)	66/91 (73)	78/91 (86)
≥60	40/49 (82)	39/49 (80)	44/49 (90)	45/49 (92)	42/49 (86)	35/49 (71)	41/49 (84)
All ages	445/668 (67)	494/668 (74)	568/668 (85)	588/668 (88)	587/668 (88)	377/668 (56)	553/668 (83)
Urban							
Age group, y							
<5	45/57 (79)	50/57 (88)	50/57 (88)	44/57 (77)	48/57 (84)	33/57 (58)	45/57 (79)
5–12	116/135 (86)	116/135 (86)	125/135 (93)	123/135 (91)	122/135 (90)	103/135 (76)	120/135 (89)
13–18	75/84 (89)	75/84 (89)	81/84 (96)	77/84 (92)	78/84 (93)	69/84 (82)	78/84 (93)
19–34	102/130 (78)	98/130 (75)	100/130 (77)	99/130 (76)	98/130 (75)	77/130 (59)	92/130 (71)
35–59	110/130 (85)	110/130 (85)	117/130 (90)	112/130 (86)	111/130 (85)	98/130 (75)	111/130 (85)
≥60	57/62 (92)	55/62 (89)	56/62 (90)	55/62 (89)	53/62 (85)	51/62 (82)	53/62 (85)
All ages	505/598 (84)	504/598 (84)	529/598 (88)	510/598 (85)	510/598 (85)	431/598 (72)	499/598 (83)

*Baseline (BD1) 20 July–17 September 2020, second draw (BD2) 21 September–10 October 2020, third draw (BD3) 23 November–12 December 2020, fourth draw (BD4) 25 January–20 February 2021, fifth draw (BD5) 22 March–11 April 2021.

Appendix Table 3. Individuals seropositive for SARS-CoV-2 antibodies with adjusted seroprevalence and 95% creditable intervals by blood draw (BD), site and age, July 2020 – April 2021, South Africa*

Characteristic	BD1	BD2	BD3	BD4	BD5
Rural					
Age group, y					
<5	0/25 (4, 0–13)	1/49 (4, 0–13)	2/78 (4, 1–9)	14/87 (17, 10–25)	15/89 (18, 10–26)
5–12	2/148 (2, 0–5)	5/169 (3, 0–5)	7/195 (4, 2–7)	29/202 (15, 10–20)	39/201 (20, 14–25)
13–18	1/69 (3, 0–7)	5/72 (8, 0–7)	8/80 (11, 5–18)	20/82 (25, 16–35)	24/78 (31, 22–42)
19–34	0/87 (1, 0–4)	5/88 (7, 0–4)	13/91 (15, 8–23)	33/91 (37, 27–47)	35/95 (37, 28–47)
35–59	1/76 (2, 0–7)	5/77 (7, 0–7)	7/80 (10, 4–17)	23/81 (29, 20–39)	27/82 (33, 23–44)
≥60	1/40 (5, 0–13)	4/39 (12, 0–13)	4/44 (11, 3–21)	8/45 (19, 9–32)	10/42 (25, 13–38)
All ages	5/445 (1, 0–2)	25/494 (5, 0–2)	41/568 (7, 5–9)	127/588 (22, 18–25)	150/587 (26, 22–29)
Urban					
Age group, y					
<5	2/45 (6, 1–15)	5/50 (11, 4–21)	7/50 (15, 7–26)	8/44 (20, 9–32)	13/48 (28, 17–41)
5–12	11/116 (10, 5–16)	17/116 (15, 9–22)	24/125 (20, 13–27)	32/123 (26, 19–34)	38/122 (31, 23–40)
13–18	14/75 (19, 11–29)	19/75 (26, 17–36)	30/81 (37, 27–48)	35/77 (46, 35–56)	41/78 (53, 42–64)
19–34	10/102 (10, 5–17)	17/98 (18, 11–26)	23/100 (23, 16–32)	29/99 (30, 21–39)	34/98 (35, 26–44)
35–59	33/110 (30, 22–39)	45/110 (41, 32–50)	52/117 (45, 36–54)	61/112 (55, 45–64)	65/111 (59, 49–68)
≥60	3/57 (7, 2–14)	6/55 (12, 5–22)	7/56 (14, 6–24)	16/55 (30, 19–42)	18/53 (35, 22–47)
All ages	73/505 (15, 12–18)	109/504 (22, 18–25)	143/529 (27, 23–31)	181/510 (36, 32–40)	209/510 (41, 37–45)

*Baseline (BD1) 20 July–17 September 2020, second draw (BD2) 21 September–10 October 2020, third draw (BD3) 23 November –12 December 2020, fourth draw (BD4) 25 January–20 February 2021, fifth draw (BD5) 22 March–11 April 2021.

Appendix Table 4. SARS-CoV-2 seroprevalence from the fifth blood collection (22 March – 11 April 2021), by site, age group and HIV status, South Africa

	Rural			Urban		
Age group, y	HIV-uninfected	HIV-infected	p-value	HIV-uninfected	HIV-infected	p-value
<5	15/87 (17)	0/1 (0)	0.65	12/46 (26)	0/1 (0)	0.55
5–12	37/195 (19)	0/2 (0)	0.49	35/116 (30)	1/3 (33)	0.91
13–18	21/66 (32)	0/3 (0)	0.24	38/73 (52)	3/4 (75)	0.37
19–34	22/65 (34)	10/23 (43)	0.41	26/73 (36)	7/19 (37)	0.92
35–59	12/36 (33)	12/39 (31)	0.81	37/61 (61)	28/47 (60)	0.91
≥60	6/27 (22)	4/10 (40)	0.28	17/46 (37)	1/7 (14)	0.24
All ages	113/476 (24)	26/78 (33)	0.07	165/415 (40)	40/81 (49)	0.11

Appendix Table 5. Comparison of participants with detectable SARS-CoV-2 antibodies at the fifth blood collection (22 March–11 April 2021), South Africa*

Characteristic	Seropositive	Multivariable aOR (95% CI)
Site		
Rural	150/587 (26)	Referent
Urban	209/510 (41)	1.9 (1.5–2.5)
Sex		
Male	130/441 (29)	Referent
Female	229/656 (35)	1.2 (0.9–1.6)
Age group, y		
<5	28/137 (20)	1.2 (0.7–1.9)
5–12	77/323 (24)	Referent
13–18	65/156 (42)	2.6 (1.5–4.5)
19–34	69/193 (36)	2.0 (1.2–3.4)
35–59	92/193 (48)	3.2 (1.8–5.5)
≥60	28/95 (29)	1.5 (0.8–2.9)
HIV status		
Negative	278/891 (31)	1.0 (0.7–1.5)
Positive	66/159 (42)	Referent

*aOR: adjusted odds ratio.

Appendix Table 6. Comparison of participants with detectable SARS-CoV-2 antibodies after the first wave (blood draw 3) and second wave (blood draw 5), by site, July 2020–April 2021, South Africa*

	Rural				Urban			
	No. (%)				No. (%)			
Age group,y	Sero+ B3	Sero+ B5	Infected wave 2	OR (95% CI	Sero+ B3	Sero+ B5	Infected wave 2	OR (95% CI)
<5	2/76 (3)	12/76 (16)	10/12 (83)	1.8 (0.3–10.6)	12/76 (16)	12/45 (27)	6/13 (46)	3.2 (0.9–11.0)
5–12	7/193 (4)	36/193 (19)	29/36 (81)	1.5 (0.5–5.1)	36/193 (19)	38/120 (32)	15/38 (39)	2.4 (1.0–5.8)
13–18	7/76 (9)	23/76 (30)	16/23 (70)	0.8 (0.2–2.9)	23/76 (30)	41/78 (53)	12/41 (29)	1.5 (0.6–3.8)
19–34	13/89 (15)	33/89 (37)	20/33 (61)	0.6 (0.2–1.7)	33/89 (37)	32/92 (35)	13/34 (38)	2.3 (0.9–5.7)
35–59	7/78 (9)	26/78 (33)	19/26 (73)	Referent	26/78 (33)	65/111 (59)	14/66 (21)	Referent
≥60	4/41 (10)	10/41 (24)	6/10 (60)	0.6 (0.1–2.6)	10/41 (24)	18/53 (34)	11/18 (61)	5.8 (1.9–17.8)

* OR: odds ratio; Sero+: seropositive.

Appendix Table 7. Comparison of individuals with and without a blood draw 3 and 5 pair, South Africa*

Characteristic	Missing 3,5 pair	Univariate OR (95% CI)
Site		
Rural	115/668 (17)	1.0 (0.8–1.4)
Urban	99/598 (17)	Referent
Sex		
M	85/503 (17)	1.0 (0.7–1.3)
F	129/763 (17)	Referent
Age group, y		
<5	38/159 (24)	2.7 (1.6–4.4)
5–12	37/350 (11)	Referent
13–18	24/178 (13)	1.3 (0.8–2.3)
19–34	66/247 (27)	3.1 (2.0–4.8)
35–59	32/221 (14)	1.4 (0.9–2.4)
≥60	17/111 (15)	1.5 (0.8–2.8)
HIV status		
Negative	112/968 (12)	Referent
Positive	22/178 (12)	1.1 (0.7–1.8)

*OR: odds ratio.

Appendix Table 8. The PHIRST-C Group

Name	Affiliation
Amelia Buys	Centre for Respiratory Diseases and Meningitis, National Institute for Communicable Diseases of the National Health Laboratory Service, Johannesburg, South Africa.
Anne von Gottberg	Centre for Respiratory Diseases and Meningitis, National Institute for Communicable Diseases of the National Health Laboratory Service, Johannesburg, South Africa. School of Pathology, Faculty of Health Sciences, University of the Witwatersrand, Johannesburg, South Africa.
Cheryl Cohen	Centre for Respiratory Diseases and Meningitis, National Institute for Communicable Diseases of the National Health Laboratory Service, Johannesburg, South Africa. School of Public Health, Faculty of Health Sciences, University of the Witwatersrand, Johannesburg, South Africa.
F. Xavier Gómez-Olivé	MRC/Wits Rural Public Health and Health Transitions Research Unit (Agincourt), Faculty of Health Sciences, School of Public Health, University of the Witwatersrand, Johannesburg, South Africa.
Floidy Wafawanaka	MRC/Wits Rural Public Health and Health Transitions Research Unit (Agincourt), Faculty of Health Sciences, School of Public Health, University of the Witwatersrand, Johannesburg, South Africa.
Jackie Kleynhans	Centre for Respiratory Diseases and Meningitis, National Institute for Communicable Diseases of the National Health Laboratory Service, Johannesburg, South Africa. School of Public Health, Faculty of Health Sciences, University of the Witwatersrand, Johannesburg, South Africa.
Jacques du Toit	MRC/Wits Rural Public Health and Health Transitions Research Unit (Agincourt), Faculty of Health Sciences, School of Public Health, University of the Witwatersrand, Johannesburg, South Africa.
Jinal N. Bhiman	Centre for Respiratory Diseases and Meningitis, National Institute for Communicable Diseases of the National Health Laboratory Service, Johannesburg, South Africa. School of Pathology, Faculty of Health Sciences, University of the Witwatersrand, Johannesburg, South Africa.
Jocelyn Moyes	Centre for Respiratory Diseases and Meningitis, National Institute for Communicable Diseases of the National Health Laboratory Service, Johannesburg, South Africa. School of Public Health, Faculty of Health Sciences, University of the Witwatersrand, Johannesburg, South Africa.
Kathleen Kahn	MRC/Wits Rural Public Health and Health Transitions Research Unit (Agincourt), Faculty of Health Sciences, School of Public Health, University of the Witwatersrand, Johannesburg, South Africa.
Kgaugelo Patricia Kgasago	Perinatal HIV Research Unit (PHRU), University of the Witwatersrand, Johannesburg, South Africa.
Limakatso Lebina	Perinatal HIV Research Unit (PHRU), University of the Witwatersrand, Johannesburg, South Africa.
Linda de Gouveia	Centre for Respiratory Diseases and Meningitis, National Institute for Communicable Diseases of the National Health Laboratory Service, Johannesburg, South Africa.
Maimuna Carrim	Centre for Respiratory Diseases and Meningitis, National Institute for Communicable Diseases of the National Health Laboratory Service, Johannesburg, South Africa.
Meredith L. McMorrow	Influenza Division, Centers for Disease Control and Prevention, Atlanta, Georgia, United States of America. Influenza Program, Centers for Disease Control and Prevention, Pretoria, South Africa.
Mignon du Plessis	Centre for Respiratory Diseases and Meningitis, National Institute for Communicable Diseases of the National Health Laboratory Service, Johannesburg, South Africa.
Neil A. Martinson	Perinatal HIV Research Unit (PHRU), University of the Witwatersrand, Johannesburg, South Africa. Johns Hopkins University Center for TB Research, Baltimore, Maryland, United States of America.
Nicole Wolter	Centre for Respiratory Diseases and Meningitis, National Institute for Communicable Diseases of the National Health Laboratory Service, Johannesburg, South Africa. School of Pathology, Faculty of Health Sciences, University of the Witwatersrand, Johannesburg, South Africa.
Retshidisitswe Kotane	Centre for Respiratory Diseases and Meningitis, National Institute for Communicable Diseases of the National Health Laboratory Service, Johannesburg, South Africa. School of Pathology, Faculty of Health Sciences, University of the Witwatersrand, Johannesburg, South Africa.
Stefano Tempia	Influenza Division, Centers for Disease Control and Prevention, Atlanta, Georgia, United States of America. Influenza Program, Centers for Disease Control and Prevention, Pretoria, South Africa. School of Public Health, Faculty of Health Sciences, University of the Witwatersrand, Johannesburg, South Africa. MassGenics, Atlanta, Georgia, United States of America.
Stephen Tollman	MRC/Wits Rural Public Health and Health Transitions Research Unit (Agincourt), School of Public Health, Faculty of Health Science, University of the Witwatersrand.
Tumelo Moloantoa	Perinatal HIV Research Unit (PHRU), University of the Witwatersrand, Johannesburg, South Africa.

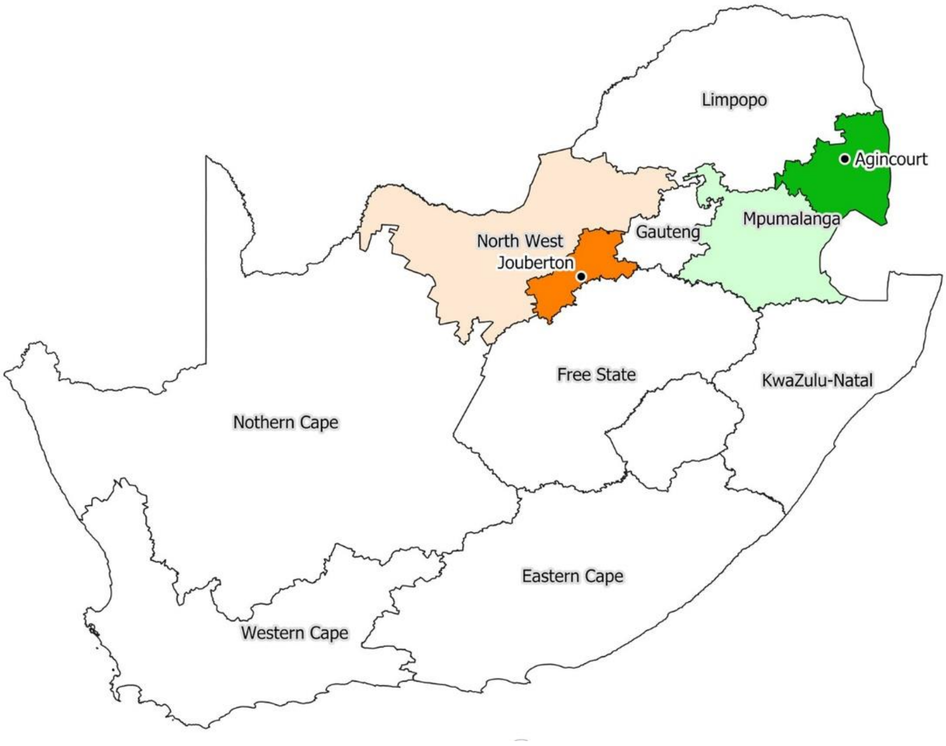

Appendix Figure 1. Location of study sites PHIRST-C, South Africa. The rural site is located in Agincourt, Ehlanzeni District (dark green), Mpumalanga Province (light green) and the urban site in Jouberton, Dr Kenneth Kaunda District (dark orange), North West Province (light orange).

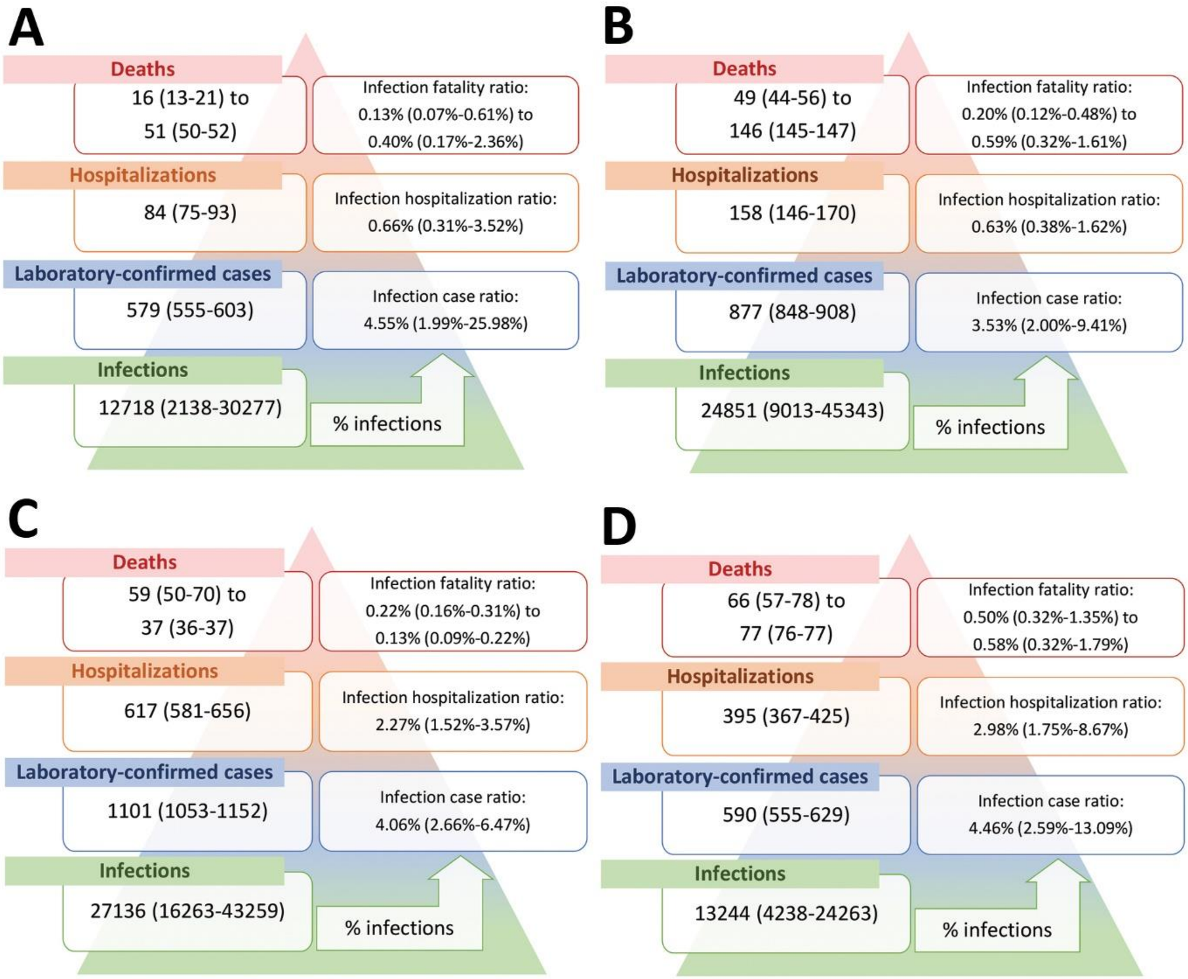

Appendix Figure 2. World Health Organization population age-standardized SARS-CoV-2 infection, diagnosis, hospitalization and deaths per 100,000 population in a rural community during A) wave 1 and B) wave 2, and urban community during C) wave 1 and D) wave 2 of infections, South Africa, March 2020–March 2021.
